# Characterization of lymphocyte populations in nonspecific interstitial pneumonia*

**DOI:** 10.1186/1465-9921-6-137

**Published:** 2005-11-15

**Authors:** Karina A Keogh, Andrew H Limper

**Affiliations:** 1 Thoracic Diseases Research Unit, Division of Pulmonary & Critical Care Medicine, Department of Internal Medicine, Mayo Clinic College of Medicine, Rochester. MN, 55905, USA

**Keywords:** Cytokines, Lymphocytes, Nonspecific interstitial pneumonitis, Pulmonary fibrosis, Usual interstitial pneumonitis

## Abstract

**Study objectives:**

Nonspecific interstitial pneumonia (NSIP) has been identified as a distinct entity with a more favorable prognosis and better response to immunosuppressive therapies than usual interstitial pneumonia (UIP). However the inflammatory profile of NSIP has not been characterized.

**Design:**

Using immunohistochemistry techniques on open lung biopsy specimens, the infiltrate in NSIP was characterized in terms of T and B cells, and macrophages, and the T cell population further identified as either CD4 (helper) or CD8 (suppressor-cytotoxic) T cells. The extent of Th1 and Th2 cytokine producing cells was determined and compared to specimens from patients with UIP.

**Results:**

In ten NSIP tissue samples 41.4 ± 4% of mononuclear cells expressed CD3, 24.7 ± 1.8% CD4, 19.1 ± 2% CD8, 27.4 ± 3.9% CD20, and 14.3 ± 1.6% had CD68 expression. Mononuclear cells expressed INFγ 21.9 ± 1.9% of the time and IL-4 in 3.0 ± 1%. In contrast, biopsies from eight patients with UIP demonstrated substantially less cellular staining for either cytokine (INFγ; 4.6 ± 1.7% and IL-4; 0.6 ± 0.3%). Significant populations of CD20 positive B-cells were also identified.

**Conclusion:**

The lymphocytic infiltrate in NSIP is characterized by an elevated CD4/CD8 T-cell ratio, and is predominantly of Th1 type, with additional populations rich in B-cells. Such features are consistent with the favorable clinical course observed in patients with NSIP compared to UIP.

## Introduction

**N**onspecific interstitial pneumonia (NSIP) has recently been identified as a distinct form of idiopathic interstitial pneumonia, distinguishable from usual interstitial pneumonia (UIP). NSIP has been associated with better response to immunosuppressive therapies and a more favorable prognosis [1-4]. Histological examination demonstrates that NSIP is characterized by a mononuclear lymphocytic interstitial infiltrate, with occasional foci of fibroblasts and variable collagen deposition [3,5]. However, the prevalence of B and T cell populations in NSIP, and specifically the CD4 or CD8 T cell content has not been fully defined in this disorder. Moreover, the relative Th1 or Th2 cytokine expression associated with this disease is also not yet known.

Inflammatory responses are generally categorized into two major types on the basis of the predominant cytokines secreted. Most autoimmune diseases, including pulmonary diseases such as sarcoidosis, follow a Th1 pattern, whereas allergic diseases such as asthma generally demonstrate a Th2 pattern [6,7]. The relevance of patterned cytokine expression during pulmonary fibrosis has been supported by a number of studies [8-11]. For instance, Th1 cells produce predominantly interferon gamma (IFNγ) and interleukin 2 (IL-2), which impair fibroblast activation and proliferation and suppress collagen production. In contrast, Th2 cells secrete IL-4, IL-10, and IL-13. Th2 cells may thereby act to stimulate fibroblast growth and promote collagen production. Thus, the relative extent of Th1 and Th2 cytokine production may underlie the tendency of various interstitial lung diseases toward more or less rapid progression, and may further limit the extent of reversibility in these disorders.

Accordingly, the following study was performed to determine the cellular populations present in lung tissue from patients with NSIP. We first characterized the infiltrate in NSIP in terms of T and B cells, and macrophages, and further identified the T cell population as either CD4 (helper) or CD8 (suppressor-cytotoxic) T cells. This was undertaken utilizing immunohistochemistry on tissues obtained by open lung biopsy. As a second aim, we determined the extent of Th1 and Th2 cytokine producing cells in lung tissues obtained from these patients with NSIP. In comparison lung tissues from patients with UIP were analyzed concurrently.

## Materials and methods

### Subjects and Tissue Collection

The Mayo Foundation Institutional Review Board approved these studies. All biopsies were obtained during the routine clinical care of these patients. The study population consisted of ten patients with a pathologic confirmation of NSIP established by experienced pulmonary pathologists. The diagnosis was based on histological findings in biopsies obtained by video assisted thoracoscopy, between November 1997 and present according to previously published criteria [3,12]. There were seven women and three men in our study population, with a mean age of 50.5 years (range 17–66) (Table 1). Three were prior smokers and seven were never smokers. All except one patient, had moderate to severe inflammation present on the biopsy. In case number 1, the inflammation was judged as mild to moderate. One patient was receiving high dose intravenous methylprednisolone at the time of biopsy (case 5), and two were receiving oral prednisone. Three additional patients had received prednisone within the six months prior to biopsy (Table 1). In preliminary studies, tonsillar tissue was used as a lymphocyte-rich control to confirm that the immunohistochemical procedures employed were robust in their ability to detect the specified cellular antigens. For comparison of Th1 (IFNγ and Th2 (IL-4) cytokine expression, samples from a group of patients with UIP were studied in parallel. This population consisted of eight patients with a clinicopathologic diagnosis of UIP and a mean age of 65.6 years (range 60–81). Four were prior smokers and four had never smoked. These UIP patients were previously reported as part of a separate, strictly morphological, study [1].

**Table 1:** NSIP Patient Characteristics

**Case**	**Age**	**Sex**	**Background Illnesses**	**Steroids prior to Biopsy**	**CD4/CD8**	Lymphoid Aggregates*	**INFγ %**	**IL-4 %**
1	50	F	-	-	1.9	-	24.5	1
2	66	F	-	Previous	1.1	++	19.8	8.2
3	66	M	-	Current	1.5	-	14	3.9
4	51	M	-	-	1.1	+	17.3	-
5	17	F	ARF†	Current	2.1	++	20.7	4.8
6	45	F		Current	0.8	+	12.8	1.1
7	65	F	Nitrofurantoin exposure	Previous	0.9	++	26	0.5
8	57	F	-	-	1.5	++	25.5	2.4
9	31	M	-	Previous	1.5	++	28.1	7.2
10	57	F	CREST	-	1.2	++	30	0.7

• Lymphoid aggregates, + = small aggregates, ++ large aggregates, - more fibrotic

• † = acute respiratory failure present clinically prior to biopsy.

### Immunohistochemical Evaluation

Formalin-fixed, paraffin embedded sections of 5-μm thickness were deparaffinized through three, 20 minute, exchanges of xylene. The tissues were then rehydrated using a graded series of alcohol washes (100%, 100%, 95%, 70%, 50% and 30%), and then incubated for 30 minutes in 0.5% hydrogen peroxide to quench endogenous peroxidase activity. After 30-minute incubation with blocking serum (1% horse serum for mouse primary antibodies and 1% goat serum for rabbit antibodies), the primary antibodies were applied. All primary antibodies were mouse monoclonal antibodies, with the exception of a CD3 rabbit polyclonal antibody. The primary antibodies evaluated were those recognizing CD3 (5 μg/ml, DAKO Corporation, Carpinteria, CA), CD4 (41 μg/ml, Novocastra Laboratories, Newcastle upon Tyne, UK), CD8 (1 μg/ml Serotec, Raleigh, NC), CD20 (8 μg/ml, DAKO) a B cell marker, CD68 (used undiluted, DAKO) a monocyte/macrophage marker, IL-4 (15 μg/ml, Stem Cell Technologies, Vancouver, BC), and INFγ (2.5 μg/ml, Stem Cell Technologies) [13]. Antibody dilutions were applied uniformly in parallel across all tissues studied. Enzymatic pretreatment for antigen retrieval was necessary for the detection of CD3 and INFγ, using Proteinase K (20 μg/ml for 10 minutes at room temperature, Invitrogen Corporation, Carlsbad, CA), and IL-4, using Protease XXV (1000 μg/ml for 10 minutes at 37°C, NeoMarkers, Fremont, CA). Heat retrieval of epitopes by boiling was used for the CD4 and CD8 studies in the presence of 1 mM EDTA; pH 8 for 10 minutes (Sigma, St Louis, MO), and in the case of CD20, utilizing 10 mM sodium citrate buffer; pH 6 for 10 minutes (Sigma). Primary antibody binding was detected using the avidin-biotin immunoperoxidase method (Vectastain Elite ABC Kit, Vector Laboratories, Burlingame, CA) with 3-amino-9-ethyl-carbazole substrate (AEC) as the colorimetric substrate, producing a red to brown pigment. The sections were counterstained with 1% hematoxylin. The percentage of positively stained cells in each sample was determined by counting stained and non-stained mononuclear cells in 5 randomly selected contiguous high-power fields (400× magnification). Fibroblasts, epithelial, endothelial cells and intravascular cells were excluded in the enumeration procedure [14]. The coefficient of variation (standard deviation/mean × 100%) of the enumeration procedure was ~17% on repeated counting of the same stained sections.

### Statistical analysis

Descriptive analyses were performed using the statistical software package, JMP version 4.0 (SAS Institute Inc., NC). Results are expressed as mean ± standard error of the mean. Differences between non-parametric groups were analyzed using Wilcoxon/Kruskal-Wallis tests. *P *< 0.05 was considered a statistically significant difference. Coefficients of variation were calculated from triplicate slide counts from nine slides recounted in a random blinded manner.

## Results

**Figure 1:**
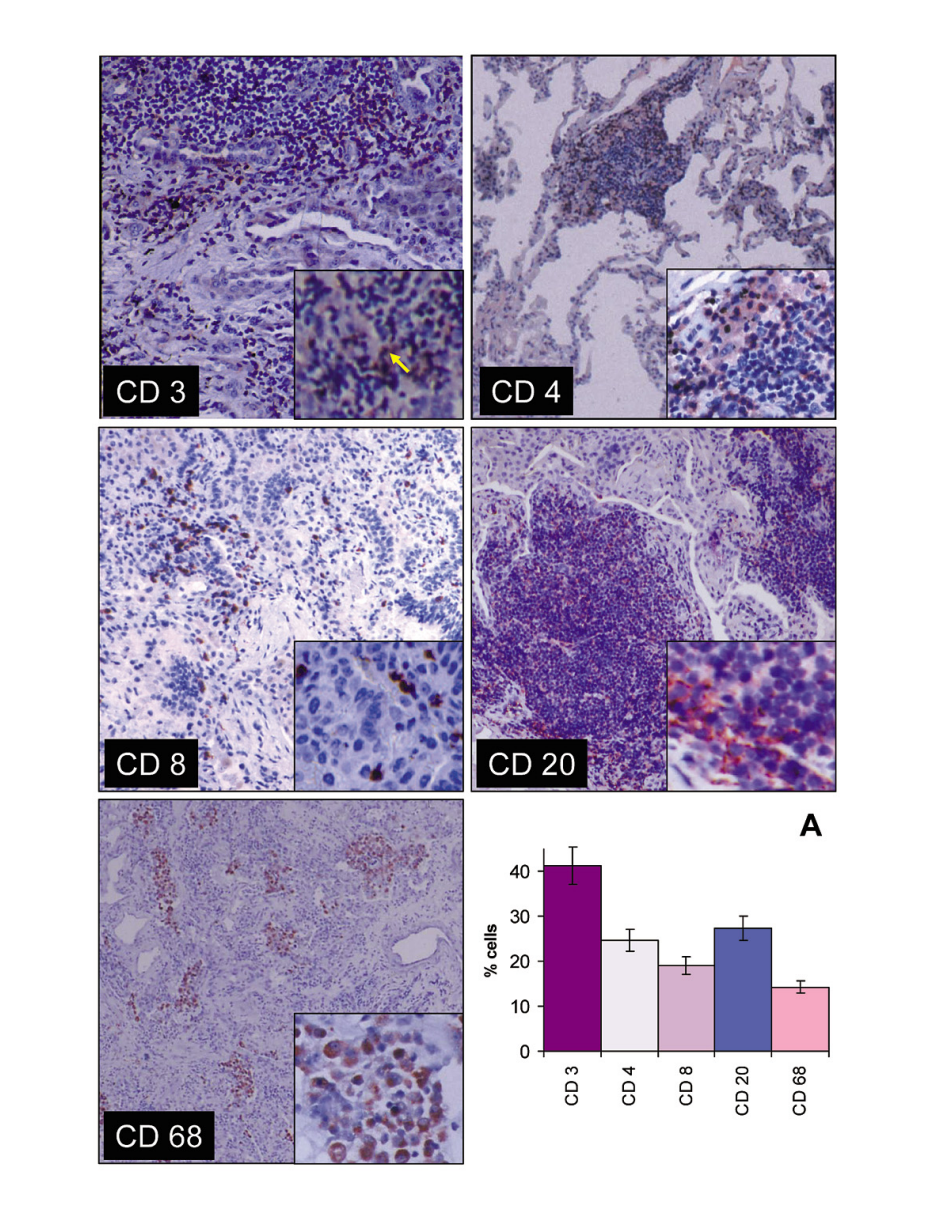
**Immunohistochemical localization of CD3, CD4, CD8, CD20 and CD68 in lung tissue with NSIP**. Bound primary antibodies to these antigens were detected by an avidin-biotin immunoperoxidase method, with AEC substrate (arrow) and counterstained with 1% hematoxylin. Staining for CD3 was present on lymphocytes located throughout the interstitium and lymphoid follicles. CD4 cells were primarily present in a perifollicular location. CD8 expressing cells were scattered throughout the interstitium. CD20 was also primarily localized to the follicles. The CD68 macrophage/monocyte marker was found in clusters of the intra-alveolar spaces and on occasional individual cells within the interstitium. **A**. Percentage of cells stained with each individual antibody, expressed as a percentage of mononuclear cells within the interstitium. Values are reported as mean ± SEM (N = 10 patients).

The NSIP tissues were rich in mononuclear cells, with a relatively high CD4/CD8 ratio, and a large number of B cells. Abundant lymphoid aggregates were seen in eight of ten specimens. Overall 41.4 ± 4.0% of interstitial mononuclear cells expressed the pan T cell surface marker, CD3 (Figure 1). These cells were found scattered throughout the interstitium and at the peripheries of lymphoid follicles. In addition 24.7 ± 1.8% of the total mononuclear cells were classified as CD4 lymphocytes (59.7% of the T cells). CD4 cells were found primarily in circumferential mantles around lymphoid follicles, but were also scattered throughout the interstitial spaces. There was also low grade staining of additional cells with this antibody, which appeared morphologically to represent alveolar macrophages. Furthermore, 19.1 ± 2.0% of the total mononuclear cell population were classified as CD8 lymphocytes (46.5% of the T cells) (Figure 1). CD8 expressing cells were found as strongly stained individual cells scattered throughout the interstitium. The CD4/CD8 ratio was 1.36 ± 0.13 (Table 1). When values from patients on steroid treatment at the time of biopsy were excluded the ratio was not found to be significantly different. In addition, the B cell marker CD20 was present on 27.4 ± 3.9% of the mononuclear cells. B cells were detected as strongly stained cells primarily within lymphoid follicles. We further observed that 14.3 ± 1.6% of mononuclear cells displayed the macrophage/monocyte marker CD68. These were localized as clusters in the airspaces and as occasional individual cells within the interstitium (Figure 1). When intra-alveolar cells were included 30.0 +/- 3.1% of all mononuclear cells expressed the CD68 antigen.

**Figure 2:**
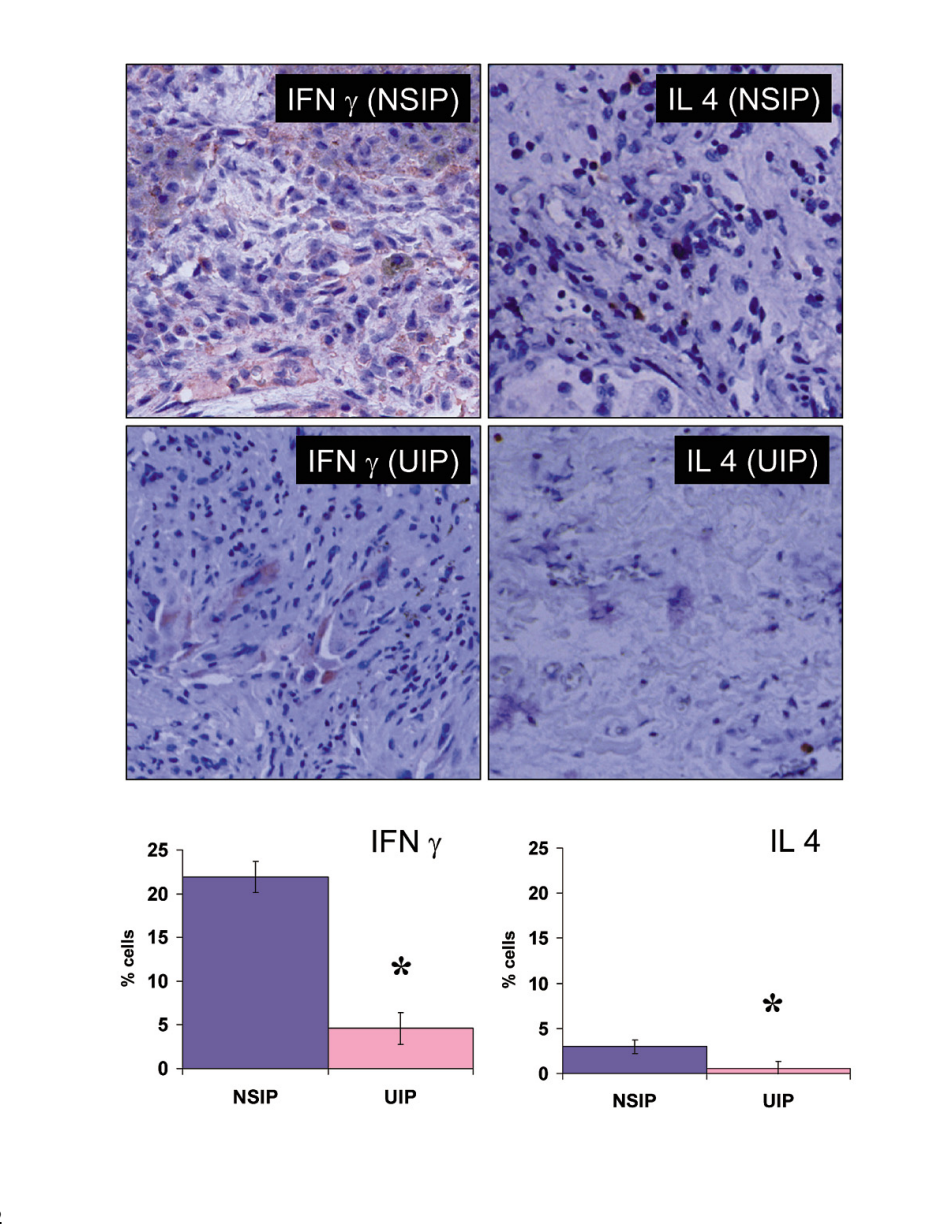
**Immunohistochemical localization of cell-associated INFγ and IL-4 in NSIP and UIP lung biopsies**. NSIP tissue exhibited diffuse expression of INFγ on lymphocytes. INFγ was also associated with macrophages and epithelial cells. Few cells stained positive for IL-4 in NSIP lung. Both stains were also significantly less prominent in UIP. The percentage of stained cells was enumerated for the NISP and UIP samples. Values are reported as mean ± SEM for 10 NSIP and 8 UIP biopsies. (* Denotes significant differences in the extent of cellular staining, for INFγ P = 0.0005, for IL-4 p = 0.045 comparing NSIP and UIP by Wilcoxon/Kruskal-Wallis Tests).

Cytokine expression was substantially greater in NSIP compared with UIP tissues. INFγ is a prototypic cytokine characteristic of a Th1 type cytokine response. In these tissues from with NSIP, cytoplasmic staining for IFNγ was demonstrated in 21.9 ± 1.9% of the interstitial mononuclear cells (Figure 2). There was also abundant staining of intra-alveolar macrophages and some staining of epithelial cells for cell associated IFNγ. In contrast, IL-4 expression is more typical of a Th2 patterned cytokine response. IL-4 was detected in 3.0 ± 1.0% of mononuclear cells in subjects with NSIP. These IL-4 expressing cells were found scattered throughout the biopsies as individual mononuclear cells. There was also some limited background staining of epithelial cells for IL-4. For comparison, 9 UIP samples were also evaluated for IFNγ and IL-4 expression. There was much less staining for either cytokine in the UIP samples (Figure 2). In the UIP biopsies, very occasional mononuclear cells (4.6 ± 1.7%) and only occasional epithelial cells displayed any cell associated INFγ. Virtually no cells exhibited IL-4 (0.6 ± 0.3%) in the UIP tissues evaluated.

## Discussion

Alveolar interstitial lymphocytes are rare in normal lung parenchyma. The presence of interstitial and alveolar lymphocytes in NSIP has been previously documented both on histology and bronchoalveolar lavage (BAL) [3,4,14,15]. This study was undertaken to further characterize the inflammatory cell infiltrate in NSIP. The key findings were; 1) The cellular infiltrate in NSIP is largely composed of lymphocytes with a relatively high CD4/CD8 ratio, 2) A large proportion of the mononuclear cells express the B cell specific antigen CD20, and 3) Cytokine expression was substantially greater in NSIP compared with UIP tissues. This cytokine expression in NSIP was predominantly a Th1 patterned response.

The observed CD4/CD8 cellular ratio of 1.36 ± 0.13 was higher than expected and is higher than has been reported in two prior studies [4,17]. One of these previous investigations evaluated only BAL data, and the other studied both BAL and histology in NSIP and pulmonary fibrosis associated with connective tissue disease. The perifollicular locations of these cells make them less likely to be washed from the alveoli during BAL [16,17]. In addition, the majority of our NSIP biopsies demonstrated a high degree of cellularity rather than fibrosis, which may have also influenced our observations. Our finding that this cellularity contains a large number of CD20 positive B cells is of interest, and may suggest a new target for treatment of NSIP. In patients who are not responding to traditional therapy, there may be a possible role for agents such as rituximab, a monoclonal antibody against CD20 [18-20].

Clinically, NSIP behaves as a more inflammatory process, with greater responsiveness to immunosuppressive therapy, in distinct contrast to UIP. Our findings of cytokine-rich infiltrates in NSIP further support these observations. In our study, the cytokine expression pattern in NSIP appears to be consistent with a predominant Th1 response. The dichotomy between Th1 and Th2 cells was first demonstrated in murine CD4 T cell clones [21]. It has since been identified in humans with chronic inflammatory lesions [22-24].

IFNγ, which is secreted from Th1 cells, has been shown in previous studies to suppress fibroblast activity *in vitro *and in murine models of bleomycin induced fibrosis [6,24-27]. Investigations have also suggested that patients with UIP have impaired production of INFγ and that the administration of IFNγ can alter their disease process [10,28]. In contrast, Th2 cells secrete IL-4, which has been implicated as a fibroblast-stimulating agent [29,30]. IL-4 has been found to be upregulated in some murine models of fibrosis [31]. Enhanced production of IL-4 has also been observed in pulmonary fibrosis associated with systemic sclerosis, which also exhibits a lower Th2/Th1 ratio than UIP, and further is associated with a substantially higher level of INFγ production in tissue [9]. The level of cell staining for IL-4 in the UIP samples in our study was somewhat lower than has previously been reported. This may have been influenced by the general lack of cellularity of our UIP samples, as UIP is associated with heterogeneous involvement of the lung parenchyma. These previous studies on UIP suggested a Th2 type response with very low levels of INFγ, and higher levels of IL-4 [9,10].

Our study indicates that there is a substantial increase in IFNγ production in NSIP when compared to both our UIP specimens and previous publications [9,10]. This increased level of IFNγ in NSIP, would be expected to counteract the postulated pro-fibrotic effect of IL-4 and may help explain the relative lack of fibrotic foci in this form of idiopathic interstitial pneumonia.

One limitation to our study is the inclusion of patients in the study group who were receiving glucocorticoids, or had been treated with them in the past. This was necessary as during this time period, at our institution, only a small number of patients with interstitial lung disease were proceeding to surgical lung biopsy without a previous trial of glucocorticoids. In analyzing the results, there was no significant difference between cytokine levels based on current or previous treatment.

In conclusion, we observed that NSIP is characterized by a largely lymphocytic infiltrate, with a high CD4/CD8 ratio, rich in cytokines, predominantly exhibiting a Th1 type response. These findings may in part explain why NSIP, in comparison to UIP, follows a slower, less fibrotic course, and is more responsive to immune modulatory therapies.

## Abbreviation list

AEC = 3-amino-9-ethyl-carbazole substrate

BAL = bronchoalveolar lavage

IFNγ = interferon gamma

IL = interleukin

NSIP = Nonspecific interstitial pneumonia

UIP = usual interstitial pneumonia

## Note

*This work was supported by funds from the Robert N. Brewer Family Foundation, and funds from the Mayo Foundation.
